# Provenance of a “sense‐sational” wait: A call for introducing sensory processing differences into diagnostic criteria for attention‐deficit/hyperactivity disorder

**DOI:** 10.1002/brb3.3501

**Published:** 2024-05-15

**Authors:** Victoria Sally Dunn, Stephanie Petty, Alison Laver‐Fawcett

**Affiliations:** ^1^ Humber Foundation NHS Teaching Trust York St John University, Lord Mayor's Walk York UK; ^2^ York St John University, Lord Mayor's Walk York UK

1

Attention‐deficit/hyperactivity disorder (ADHD) is predominantly understood as a neurodevelopmental disorder (Scandurra et al., 2019), which has been renamed and refined with progressive versions of the Diagnostic and Statistical Manual of Mental Disorders (DSM). In this paper, we advocate for further research into sensory processing differences experienced by people with ADHD and a possible future revision to diagnostic criteria to recognize sensory processing differences. The DSM‐II (American Psychiatric Association, APA, 1968) first presented ADHD as a “hyperkinetic reaction of childhood,” with “excessive motor activity” as a prominent symptom (Epstein & Loren, 2013, pp. 455). Between the DSM‐II and DSM‐5 Text Revision (DSM‐5‐TR, APA, 2022), re‐conceptualizations of ADHD have been transformed. Each revision has had implications for sociological understanding and consequent diagnostic decision making, with the application of new criteria (Kooij et al., 2019; Sibley et al., 2013) and expanding awareness of diagnostic overshadowing (Hatch et al., 2023). The shifting lens through which individuals view their condition is subject to changes beyond their control (Honkasilta & Koutsoklenis, 2022). It has been proposed that when the conceptualization of a condition alters so dramatically as to change its diagnostic criteria, this creates the potential to change people's life paths (Cooper, 2018).

ADHD currently has three distinct presentations, including predominantly inattentive, hyperactive, or combined inattentive and hyperactive‐impulsive types (DSM‐5‐TR, APA, 2022). Over time, symptom descriptors have been expanded and symptom thresholds have been reduced (Lange et al., 2010). These changes partly reflect a shifting acknowledgment that characteristics of ADHD are present across the lifespan (Kooij et al., 2019). In addition, there has been increased recognition that ADHD sometimes co‐occurs with autism (Coghill & Seth, 2011; Rong et al., 2021). These changes highlight the importance of robust diagnostic identification of what characterizes ADHD (Pehlivandis et al., 2021), alongside essential re‐conceptualization of ADHD based on lived experiences (Eccleston et al., 2019).

A systematic review of 11 studies conducted in 2011 concluded that “sensory processing problems in children with ADHD are more common than in typically developing children” (Ghanizadeh, 2011, p. 89). Later research presents complementary evidence for significant sensory processing differences for children with ADHD and the implications for support options (Lane & Reynolds, 2019; Shimizu et al., 2014). Personal accounts expand the emerging research literature. People with ADHD are reporting sensory processing challenges and are seeking advice and support for managing these (for example, Attention Deficit Disorder Association, ADDA, 2023; Burch, 2023; Maguire, 2021). However, online literature written to support individuals with ADHD and their families offers information of variable quality and credibility, including unreferenced recommendations with unclear authorship (Yeung et al., 2022). Furthermore, people are being directed to online assessments (for example, ADDitude Editors, 2019), which may not provide essential information about the validity of the tests or the processes of standardization (Neff, n.d).

As sensory processing differences are now recognized for autistic people (Patil & Kaple, 2023), a challenge for practitioners is how to accurately formulate the sensory needs of people with ADHD whilst minimizing additional assessments and potential co‐occurring diagnoses. Neurodivergent people frequently receive multiple diagnostic labels that overlap to explain underpinning characteristics (Kooij et al., 2019; Werkhoven et al., 2022), which has negative implications for resource management of waiting lists, risks associated with delayed pharmacological and/or nonpharmacological interventions, and difficulty managing patients’ expectations (Al‐Khudairi et al., 2019). When individuals do not receive the appropriate support for ADHD‐related difficulties because of delayed assessment or delayed treatment, identified risks have included increased harm to self, substance misuse, unemployment, and criminality (Assayag et al., 2022; Johnson et al., 2021). Improving the specificity and sensitivity of the diagnostic criteria for ADHD through revisions and refinements should enable the right diagnosis, at the right time. Sensory processing differences understood as being ubiquitous and lifelong would also contribute to how co‐occurring emotional distress is approached, including, for example, where links have been identified between sensory processing differences and anxiety and depression (Lane & Reynolds, 2019; Paquet et al., 2022).

As healthcare services experience increasing demand that often outstrips capacity (Cahill, 2023), valid and reliable diagnostic criteria remain the backbone of clinical decision‐making. The DSM‐5‐TR (APA, 2022) and International Classification of Diseases Eleventh Revision (ICD‐11; World Health Organisation, 2019) must, therefore, fairly represent all individuals with ADHD and the diversity of their clinical profiles. Any amendment to diagnostic criteria or changes in service provision must be supported by research evidence and consultation with key stakeholders (Kaap & Ne'eman, 2020; NHS England, 2022; Young et al., 2021). A paradigm shift towards neurodiversity affirmative practice is also an important point of reference, whereby differences are validated, positive neurodivergent identity is supported, and self‐advocacy is foregrounded (Pellicano & Houting, 2022).

Through examining the process and rationale used to evidence the inclusion of sensory processing differences as part of the diagnostic criteria for Autism Spectrum Disorder (ASD), it was hoped a path would be outlined for expediting this process, if appropriate, for revising the criteria for ADHD. Between the DSM‐IV and DSM‐5 (Rosen et al., 2021) ASD became an umbrella term, replacing previous diagnostic categories including Asperger's Syndrome, Childhood Disintegrative Disorder, Pervasive Developmental Disorder, and Autistic Disorder. These changes, as discussed by Cooper (2018), had significant implications for access to support services (Kent *et al.*, 2013) and for how individuals and society adjusted to the changed representation of being autistic (Gensler, 2012).

In February 2011, sensory processing sensitivities were recognized within diagnostic criteria for ASD (APA, 2010b). The DSM‐5 revision process aimed to: “use evidence from clinical practice and existing epidemiological, neurobiological, clinical, and genetics literature to develop revised or new diagnostic criteria that better capture[s] the various mental disorders to help clinicians provide more accurate diagnoses,” (Clarke et al., 2013, p. 43). Unlike the transition from DSM‐III to DSM‐IV, which published four “sourcebooks” (Widiger et al., 1998) containing archived information about the revision process, the DSM‐5 source materials do not provide this detail.

As a team of practicing clinicians and researchers, we found it difficult to identify the process followed through which sensory processing differences were recognized as a characteristic of being autistic, through to their inclusion in diagnostic criteria. Literature searches did not identify evidence sources and seeking insights from the DSM‐5 Neurodevelopmental workgroup documentation was not fruitful. An internet archive search, using Wayback Machine, provided access to the DSM‐5 Development website (APA, 2010a) and identified the only reference to the rationale for including sensory processing differences in the revised ASD criteria. It stated that the revision to recognize sensory processing differences within diagnostic criteria was based on “literature review, expert consultations, and workgroup discussions; confirmed by the results of secondary analyses of data … ” (APA, 2010c; Rationale tab 2, bullet point 5).

Acknowledging that sensory processing differences are part of being autistic has taken decades and opened the door to alternative intervention options that focus on people's individual functional needs. For instance, Schaaf et al. (2014) reported that families of autistic children who participated in a sensory integration program described reduced dependence in self‐care and social activities. Different approaches are evident for a wide range of priority needs for autistic people, such as providing school‐based support (Pastor‐Cerezuela *et al.*, 2020) or interventions for eating difficulties (Nimbley et al., 2022). The addition of sensory processing differences in the diagnostic criteria for ADHD might similarly provide alternative support options.

Clinical research with people who have ADHD has primarily reflected on issues of mis‐ and over‐diagnosis, financial implications of inaccurate diagnosis, possible reasonable adjustments to support individuals with ADHD, and pharmacological interventions (Sibley & Kuriyan, 2016). We advocate that clinicians and academics should note the emerging evidence of sensory processing differences for people with ADHD and should contribute to further exploration of their place within diagnostic criteria. The benefits of including sensory processing differences within diagnostic criteria may be threefold. First, this would ensure that people presenting with sensory processing differences are not erroneously excluded from an assessment of ADHD. Second, resources would be saved from unnecessary assessments of other neurodivergent and mental health conditions. Finally, individuals with ADHD and sensory processing differences that are affecting daily functioning could receive the tailored support needed. Personal interest and academic interest in improving understanding of sensory processing differences for people with ADHD is clear. The potential cost of overlooking their place in diagnostic criteria is high.

We suggest that it would be wise to learn from the process of introducing sensory processing differences into the diagnostic criteria for ASD in order to engage in a timely review of all available stakeholder perspectives and research evidence that represents sensory processing differences as a characteristic of ADHD.

## FUNDING INFORMATION

The first author's PhD studies are funded by a York St John Fee Scholarship for post‐graduate research course, from the Institute of Health and Care Improvement.

## CONFLICT OF INTEREST STATEMENT

The authors declare no conflict of interest.

### PEER REVIEW

The peer review history for this article is available at https://publons.com/publon/10.1002/brb3.3501.

## PATIENT CONSENT STATEMENT

Patient consent statement was not required for this paper.

## PERMISSION TO REPRODUCE MATERIAL FROM OTHER SOURCES

All sources of information used are cited and referenced.

## CLINICAL TRIAL REGISTRATION

Not applicable.

## Data Availability

Data sharing is not applicable to this paper as no datasets were generated or analyzed in the production of this paper.
